# Neuronal markers are expressed in human gliomas and NSE knockdown sensitizes glioblastoma cells to radiotherapy and temozolomide

**DOI:** 10.1186/1471-2407-11-524

**Published:** 2011-12-20

**Authors:** Tao Yan, Kai Ove Skaftnesmo, Lina Leiss, Linda Sleire, Jian Wang, Xingang Li, Per Øyvind Enger

**Affiliations:** 1 Department of Neurosurgery, Qilu Hospital, Shandong University, Jinan, P.R. China; 2 Oncomatrix Research Lab, Department of Biomedicine, University of Bergen, Norway; 3 Brain Science Research Institute, Shandong University, Jinan, P.R. China; 4 Translational Cancer Research Group, Department of Biomedicine, University of Bergen, Norway; 5 Department of Neurosurgery, Haukeland University Hospital, Bergen, Norway

## Abstract

**Background:**

Expression of neuronal elements has been identified in various glial tumors, and glioblastomas (GBMs) with neuronal differentiation patterns have reportedly been associated with longer survival. However, the neuronal class III β-tubulin has been linked to increasing malignancy in astrocytomas. Thus, the significance of neuronal markers in gliomas is not established.

**Methods:**

The expressions of class III β-tubulin, neurofilament protein (NFP), microtubule-associated protein 2 (MAP2) and neuron-specific enolase (NSE) were investigated in five GBM cell lines and two GBM biopsies with immunocytochemistry and Western blot. Moreover, the expression levels were quantified by real-time qPCR under different culture conditions. Following NSE siRNA treatment we used Electric cell-substrate impedance sensing (ECIS) to monitor cell growth and migration and MTS assays to study viability after irradiation and temozolomide treatment. Finally, we quantitated NSE expression in a series of human glioma biopsies with immunohistochemistry using a morphometry software, and collected survival data for the corresponding patients. The biopsies were then grouped according to expression in two halves which were compared by survival analysis.

**Results:**

Immunocytochemistry and Western blotting showed that all markers except NFP were expressed both in GBM cell lines and biopsies. Notably, qPCR demonstrated that NSE was upregulated in cellular stress conditions, such as serum-starvation and hypoxia, while we found no uniform pattern for the other markers. NSE knockdown reduced the migration of glioma cells, sensitized them to hypoxia, radio- and chemotherapy. Furthermore, we found that GBM patients in the group with the highest NSE expression lived significantly shorter than patients in the low-expression group.

**Conclusions:**

Neuronal markers are aberrantly expressed in human GBMs, and NSE is consistently upregulated in different cellular stress conditions. Knockdown of NSE reduces the migration of GBM cells and sensitizes them to hypoxia, radiotherapy and chemotherapy. In addition, GBM patients with high NSE expression had significantly shorter survival than patients with low NSE expression. Collectively, these data suggest a role for NSE in the adaption to cellular stress, such as during treatment.

## Background

Most primary brain tumors are classified as gliomas based on the tumor cells' resemblance with glial cell types and immunohistochemical characteristics, particularly the expression of glial fibrillary acidic protein [1,2]. However, the cellular origin of gliomas is still controversial [3,4] and the term "glioneuronal tumor" refers to gliomas which contain neuronal elements such as neurocytes or ganglion cells. In addition, several neuronal antigens have been detected in glioblastoma multiforme (GBM) [5-7], diffuse astrocytomas [8,9], pleomorphic xanthoastrocytomas [10], oligodendrogliomas [11] and ependymomas [12]. Expression of neurofilament protein (NFP) and microtubule-associated proteins in GBM cell lines have been described [13], and increasing immunoreactivity for the class III β-tubulin has been associated with an ascending gradient of malignancy [8]. Several studies have explored the relationship between neuronal antigens and the prognosis of gliomas. Varlet et al [6] reported that a GBM subclass coexpressing glial fibrillary acidic protein and neurofilament protein had a lower recurrence rate at the primary site and a better prognosis. Furthermore, prognostic subclasses of high grade gliomas have been identified, including a proneural group expressing neuronal lineage markers that was associated with longer survival than the other subgroups [3]. However, one recent study found that the prognostic significance of neuronal marker expression was limited to giant cell GBMs expressing two or more neuronal markers, and that the expression of these markers was associated with shorter survival [5]. Thus, the role of neuronal markers in human gliomas is still unclear.

In this work, we investigated the expression and regulation of four neuronal markers class III β-tubulin, neurofilament protein (NFP), microtubule-associated protein 2 (MAP2) and neuron-specific enolase (NSE) in GBM cell lines and patient biopsies. Furthermore, we quantified their expression under different culture conditions. Since NSE was consistently upregulated under different stress conditions we performed knock-down experiments using NSE siRNA to see how this impacted on glioma cell behavior. Finally, we correlated immunopositivity for NSE with patient survival using a panel of glioma biopsies.

## Methods

### Cell culture

The human GBM cell line GaMG was established at the Gade Institute, University of Bergen, Norway [14]; U87, LN229, A172, U251 were purchased from the American Tissue Culture Collection (ATCC; Manassas, VA, USA) and cultured in DMEM (Sigma, St. Louis, MO) containing 10% fetal bovine serum, supplemented with NEAA, 100 U/ml Pen/Strep, 400 μM L-glutamine, all from Cambrex (Cambrex, East Rutherford, NJ) and in stem cell medium (SCM) consisting of Neurobasal Medium (Invitrogen, Carlsbad, CA) supplemented with 20 μl/ml B27 (Invitrogen), 10 μl/ml Glutamax (Invitrogen), 20 ng/ml EGF (Sigma), 20 ng/ml FGF2 (R&D Systems, Minneapolis, MN) and 100 U/ml Pen/Strep (Cambrex). Cells were also cultured in serum-free starvation medium consisting of DMEM (Sigma) supplemented with NEAA, 100 U/ml Pen/Strep and 400 μM L-glutamine (Cambrex). The medium was changed every 2 days.

### Patient biopsies

Patient biopsies were obtained from the Department of Neurosurgery, Haukeland University Hospital, Bergen, Norway. Collection of tumor biopsies and the corresponding clinical data was appoved by the Regional Ethical Committee (REK Vest). Prior to harvesting the biopsies and clinical data, informed and written consent was obtained from each patient that provided tissue. Biopsy spheroids were prepared as previously described [15], and monolayer cells from spheroids were cultured both in DMEM medium and SCM.

### Immunocytochemistry (ICC) and immunohistochemistry (IHC)

Cell suspensions were plated on 12-mm cover slips coated with poly-L-Lysine (50 μg/ml, 2 hr at 37°C). The cells were fixed with 4% PFA for 10 min followed by 4 min permeabilization in 0.5% Triton-X at room temperature, washed 4 times in PBS and then blocked with protein block (PBS with 0.5% BSA) solution for 15 min. Primary antibodies diluted in protein block solution were incubated for 45 min at 37°C or overnight at 4°C. After three washes with PBS, fluorescent conjugated secondary antibodies were applied in combination with protein block solution for 45 min at 37°C. Cover slips were washed four times with PBS, mounted with Vectashield containing DAPI (Vector Laboratories, Burlingame, CA) or Prolong Gold antifade reagent with DAPI (Invitrogen, Carlsbad, CA) and inspected under a Nikon Eclipse TE2000-E fluorescence microscope (Nikon, Tokyo, Japan).

The primary antibodies were diluted as follows: chicken polyclonal anti-class III β-tubulin, 1:200 (Abcam, Cambridge, UK); mouse monoclonal anti-neurofilament 70 kDa, 1:50 (Chemicon, Tamecula, CA, USA); chicken polyclonal anti-MAP2, 1:100 (Abcam); rabbit polyclonal anti-NSE, 1:100 (Abcam). The secondary antibodies were diluted as follows: goat anti-chicken IgY-TR, 1:800 (Santa Cruz Biotechnology, CA, USA); goat anti-rabbit IgG, 1:800 (Southern Biotech, Birmingham, Alabama, USA); goat anti-mouse IgG_1_, 1:200 (Southern Biotech).

For IHC, paraffin sections were rehydrated through a procedure of 2 × 5 min in Xylene, 2 × 3 min in 100% EtOH, 2 × 3 min in 96% EtOH and finally in ddH_2_O for 5 min. Heat induced epitope retrieval (HIER) was done by heating sections to 95°C for 25 min in 10 mM Na-Citrate buffer pH = 6. The sections were blocked with peroxidase block buffer (DAKO, Denmark) for 5 min, washed three times with TBS Tween 0.05% and blocked with protein block (DAKO) for 30 min at room temperature. Then the sections were incubated with the rabbit anti-NSE antibody (Abcam) diluted 1:200 in buffer (25 mM Tris-HCl, 75 mM NaCl, 1% BSA, pH 7.4) overnight at 4°C, washed three times with TBS Tween 0.05% and incubated with HRP labeled polymer anti-rabbit secondary antibody (DAKO) for 40 min at room temperature. After 4 washes, the sections were developed with DAB+ (Dako) for 4 min, counterstained with haematoxylin, dehydrated and mounted with Entellan (Merck KGaA, Darmstadt, Germany). The sections were examined and photographed using a light microscope equipped with a digital camera (DXM1200, Nikon, Japan).

For each slide, ten different microscopic fields were selected and photomicrographs were captured at a magnification of 40×. Immunoreactivity was scored as area fractions calculated as the ratio of immunopositive area to the total area of the microscopic field by an investigator as described before using a morphometry software (NIS-Elements BR 3.2, Nikon, Japan) [16]. Immunostaining intensity higher than average background was set as pixel threshold for positive staining, which included both nuclear and cytoplasmic stainings. The investigator was blinded to the patient identities, with no prior knowledge of clinical and pathological parameters. Only after the scoring was completed, a clinician retrieved the patient data from the journals at the hospital.

### Immunoblots

Cultured cells were washed in PBS 2 × and subsequently homogenized in lysis buffer (20 mM MOPS, 5 mM EDTA, 2 mM EGTA, 30 mM NaF, 0.5% Triton X, 40 mM b-Glycerophosphate, 20 mM Na-Pyrophosphate, 1 mM Na-Orthovanadate, 3 mM Benzamidine, 5 uM Pepstatin, 10 uM Leupeptin, 1 mM PMSF, pH 7.2) by sonication 3 × 2 sec using Sonics Vibra Cell ^TM ^(Cole-Parmer Instruments, Vernon Hills, IL). Whole lysate was centrifuged for 30 min at 13,000 rpm and used for the subsequent analysis. Protein (10-20 μg) was added in each well and run on SDS-PAGE using NuPage precast Gels (Invitrogen). After blotting the nitrocellulose membrane for 80 min and subsequent treatment with blocking solution (TBS with 0.1% Tween, 5% milk powder) for 1 hour at room temperature the membrane was incubated overnight at 4°C in blocking solution containing chicken anti-class III β-tubulin (Abcam) diluted 1:25000, chicken anti-MAP2 (Abcam) diluted 1:100000, rabbit anti-NSE (Abcam) diluted 1:1000, mouse anti-NFL (Chemicon) diluted 1:1000 and rabbit anti-GAPDH (Abcam) diluted 1:2000. The primary antibodies were detected using a horseradish peroxidase (HRP)-conjugated rabbit anti-chicken secondary antibody diluted 1:3000 (Abcam), goat anti-rabbit secondary antibody diluted 1:100000 (Beckman Coulter), goat anti-mouse secondary antibody diluted 1:2500 (Santa Cruz Biotechnology). The Western blot was developed using Supersignal West Femto Maximum Sensitivity Substrate (Pierce Biotechnology, Rockford, IL) and detected with Fuji LAS 3000 Imager (Fuji Photo Film, Tokyo, Japan). Densitometric analysis was performed using Multi Gauge V2.3 software (Fuji Photo Film, Tokyo, Japan) and the levels of NSE were normalized to GAPDH levels.

### Isolation of total RNA

Total RNA was extracted using RNeasy Mini Kit (Qiagen GmbH, Hilden, Germany). The cell lines were washed twice in PBS, added RLT buffer and scraped off using a cell scraper. The remaining procedure was performed according to the manufacturer's instructions, including treatment with DNase I (Qiagen GmbH, Hilden, Germany).

### Real-time qPCR

250 ng total RNA was reverse transcribed in a total volume of 10 μl using iScript^TM ^cDNA Synthesis Kit (BioRad Laboratories, Hercules, CA) according to the manufacturer's instructions. The resulting cDNA reaction mix was then diluted 20 times in ddH_2_O. Real time qPCR was subsequently performed using iQ SYBR Green Supermix (Bio-Rad Laboratories) according to the manufacturer's instructions, using 0.5 μl of the diluted cDNA reaction mix in a total volume of 5 μl. The following parameters were used for the qPCR reaction: initial denaturation at 95°C for 3 min, 45 cycles of 20 sec at 95°C, 20 sec at 58°C and 20 sec at 72°C using Light Cycler 480 (Roche). Amplicon purity and size were verified by melt curve analysis. Primers directed against GAPDH were used as an internal control. Primers were designed using OligoPerfect™ Designer (Invitrogen). Human-specific primers were as follows:

Class III β-tubulin forward 5'-GCGAGATGTACGAAGACGAC-3', reverse 5'-TTTAGACACTGCTGGCTTCG-3'; NFL forward 5'-GAGGCAGCTGAAGAGGAAGA-3', reverse 5'-AAGGAAATGGGGGTTCAATC-3'; NFM forward 5'-AAGGGATCCAGGAAGGAAGA-3', reverse 5'-TGACAACGCCTTTCTCCTCT-3'; NFH forward 5'-GAGGAACACCAAGTGGGAGA-3', reverse 5'-TTCTGGAAGCGAGAAAGGAA-3'; MAP2 forward 5'-CCAATGGATTCCCATACAGG-3', reverse 5'-TCCTTGCAGACACCTCCTCT-3'; NSE forward 5'-CTGATGCTGGAGTTGGATGG-3', reverse 5'-CCATTGATCACGTTGAAGGC-3'; GAPDH forward 5'-GAGTCAACGGATTTGGTCGT-3', reverse 5'-GACAAGCTTCCCGTTCTCAG-3'. Negative controls were performed without reverse transcriptase in the reaction. In those cases, no amplification was found, ruling out contamination of the RNA samples with genomic DNA (data not shown). Fold changes were calculated using the comparative CT (2^-ΔΔCT^) method.

### RNA interference

ENO2 (NSE) silencing siRNA and nonsilencing control siRNA were purchased from Ambion. Two validated silencer select siRNAs targeting ENO2 at exon 7 or 9 were used separately in the experiment. SiRNA duplex (s4685, Ambion, Foster City, CA, USA) with sense senquence: 5'-GGUGCAGAGGUCUACCAUATT-3' and antisense sequence: 5'-UAUGGUAGACCUCUGCACCTA-3' targets exon 7. SiRNA duplex (s4684, Ambion) with sense sequence: 5'-GUGACCAACCCAAAACGUATT-3' and antisense sequence: 5'-UACGUUUUGGGUUGGUCACTG-3' targets exon 9. Cells were transfected with siRNA oligonucleotides by using Lipofectamine RNAiMAX (Invitrogen) according to the manufacturer's protocol.

### Wounding experiment using Electric cell-substrate impedance sensing (ECIS)

In ECIS, the cells are grown on the surface of small and planar gold-film electrodes, then the AC impedance of the cell-covered electrode is measured continuously at a frequency of 64 kHz. Due to the insulating properties of cell membranes, the impedance increases with increasing coverage of the electrode until a confluent layer of cells is established. 40000 A172 glioma cells were seeded out in an 8E1W ECIS array (Applied Biophysics, Troy, NY, USA), each well containing a single 250μM electrode in the middle. Cells in four of the wells were treated with 20nM NSE siRNA, whereas two wells were treated with scrambled siRNA at the same concentration (both from Ambion, Foster City, CA, USA). Upon confluence a high field current with frequency 48 kHz and amplitude 5 was applied for 10 seconds which killed cells overgrowing the electrode, creating "wounds" in the wells devoid of cells. Successful wounding was confirmed with a rapid drop in impedance and absence of cells on the electrode. Migration of surrounding glioma cells into the area overlying the electrode could then be monitored by measuring changes in impedance over time. The migration was compared between control and knockdown groups at the end of each experiment, which was presented as the ratio of impedance before and after the wounding until the end of each experiment. The experiments were performed 3 times.

### MTS assays and hypoxia, irradiation and temozolomide (TMZ) treatment

MTS assays (Promega) were performed as previously described [17]. Briefly A172 and U251 cells were seeded into 96-well plates at a density of 2000 per well and were subsequently treated with NSE or control siRNA. 18 hr after the preparation, the plates were incubated under 0.5% oxygen in a hypoxia chamber (BioSpherix, Lacona, New York, USA) or radiated with 4 Gy or treated with 10 μM (A172) or 50 μM (U251) TMZ (Tocris Bioscience, UK) and cultured in a standard tissue culture incubator with 5% CO_2 _in air and 100% relative humidity at 37°C. Each treatment condition was tested in 30 wells, excluding wells at the edge. 5 or 6 days after treatment, medium was removed and fresh medium with MTS substrate (Promega) was added to each well. Following 3 hr of incubation with MTS, the absorbance at 490 nm was measured for each well with a scanning multiwell spectrophotometer (Biochrom Asys UVM 340 Microplate Reader; Biochrom Ltd, UK). The experiments were performed 3 times.

### Patient data

The study population consisted of 28 consecutive glioma patients admitted to the Department of Neurosurgery, Haukeland University Hospital, Bergen, Norway, between January 2005 and December 2007. These patients were diagnosed with different glioma types and patient characteristics and treatment parameters are provided in Table 1.

**Table 1:** Glioma patients listed according to the NSE expression level

Patients	Diagnosis	Age	Gender	Radiotherapy(Gy)	Chemotherapy
1	GBM	58	M	60	T
2	Oligo (III)	59	M	54	T+PCV
3	Oligoastro(II)	33	M	54	T
4	GBM	66	M	36	T
5	GBM	58	M	60	T
6	GBM	58	M	60	T
7	Astro (II)	49	M	54	T
8	Astro (II)	41	M	-	-
9	GBM	42	M	60	T
10	GBM	58	F	60	T
11	GBM	67	F	60	T
12	Astro (II)	33	M	54	T+PCV
13	GBM	38	M	90	T+PCV+GK
14	GBM	22	F	60	T+PCV
15	Oligo (III)	59	M	54	T+PCV
16	GBM	58	F	76	T
17	GBM	67	M	39	-
18	GBM	67	F	60	T
19	GBM	82	M	39	-
20	GBM	55	M	60	T
21	GBM	56	M	76	T
22	GBM	74	F	60	-
23	GBM	48	M	58	T
24	GBM	66	F	60	T
25	GBM	71	F	-	-
26	GBM	58	M	60	T+GL
27	GBM	64	M	60	T
28	Oligo (II)	49	M	54	T

Oligo, oligodendroglioma; Oligoastro, oligoastrocytoma; Astro, astrocytoma; GBM, glioblastoma multiforme; F, female; M, male; T, temozolomide; PCV, procarbazine, CCNU and vincristine; GK, gamma knife surgery; GL, glivec; -, no treatment.

### Statistical analysis

Student's t-test was performed using a 2-tailed distribution analysis. The duration of survival for patients with gliomas was measured from the time of diagnosis to the time of death or last follow-up. Survival data were analyzed using the log-rank test.

## Results

### Neuronal markers are expressed in both GBM cell lines and GBM xenografts

**Figure 1:**
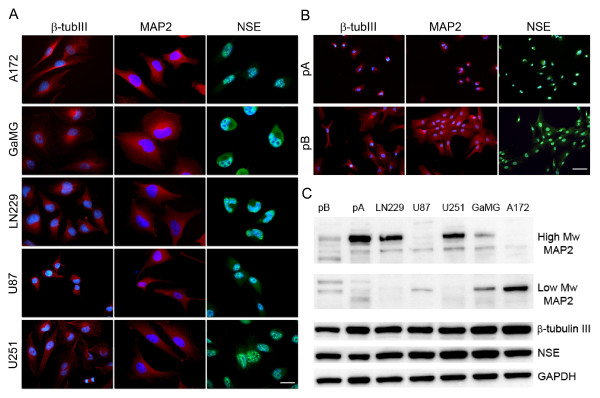
**Expression of class III β-tubulin, MAP2 and NSE in GBM cells**. **(A, B) **ICC for class III β-tubulin, MAP2 and NSE in GBM cell lines and patient specimens. Staining intensity for NSE was stronger in the nuclei than in the cytoplasms. **(C) **Western blot for class III β-tubulin, MAP2 and NSE in GBM cells. Different isoforms of MAP2 were expressed in different GBM cells. GAPDH was the loading control. High Mw MAP2, high molecular MAP2; low Mw MAP2, low molecular MAP2. Scale bars: panel A = 20 μm, panel B = 50 μm.

We performed ICC and found that NSE, class III β-tubulin and MAP2 were uniformly expressed in all the 5 GBM cell lines (A172, U251, U87, LN229 and GaMG, figure 1A) and the 2 patient biopsies (pA and pB, figure 1B). In contrast, neurofilament protein was neither detected in any of the cell lines nor the biopsies (data not shown). Immunoreactivity for NSE was observed in both the cytoplasm and the nuclear region, although it was stronger in the nuclear area than in the cytoplasm. The results were confirmed by Western blot (figure 1C). GBM cells displayed immunoreactivity to different MAP2 isoforms on the Western blot. The high-molecular weight form of MAP2 (hmw-MAP2) was mostly found in U251, LN229 and pA, while high expression of the low-molecular weight form (lmw-MAP2) was found in A172. GaMG, U87 and pB were positive for both isoforms but showed relatively weaker signals.

### NSE is upregulated under cellular stress conditions

**Figure 2:**
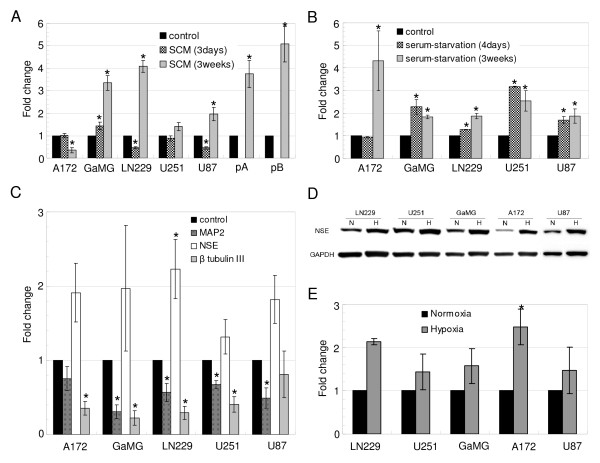
**NSE is upregulated under different cellular stress conditions**. **(A) **NSE expression assessed by real-time qPCR in 5 GBM cell lines and 2 patient specimens cultured in SCM and in **(B) **serum-starvation medium. **(C) **NSE, MAP2 and class III β-tubulin expression in GBM cell lines cultured in hypoxia, assessed by real-time qPCR. **(D) **NSE expression in 5 GBM cell lines was assessed by Western blot after 3 weeks' incubation in 0.5% oxygen hypoxia chamber. GBM cells cultured in normoxia were used as controls. **(E) **Densitometric analysis of Western blots showing increased expression of NSE in all GBM cell lines in hypoxia. mRNA and protein expression was normalized using the GAPDH gene and protein, respectively. Error bars represent SEM. Experiments were performed three times. *P< 0.05. N, normoxia; H, hypoxia.

Since real-time qPCR is a sensitive quantification method, we used this method to quantify expression of neuronal markers under different culture conditions. We used cultures of the same cell lines/biopsies in serum-containing DMEM medium in a standard tissue culture incubator with 5% CO_2 _in air and 100% relative humidity at 37°C as a reference. Furthermore, we evaluated the influence of both short-term (3 or 4 days) and long-term (21 days) incubation in SCM and serum-starvation medium on the expression of neuronal markers. After 3 days culture in SCM, NSE was downregulated in 2 of the 5 GBM cell lines (fold change in LN229, 0.5 ± 0.05, p < 0.001; U87, 0.5 ± 0.04, p < 0.001) and slightly upregulated in GaMG (1.4 ± 0.17, p = 0.041, figure 2A). After long-term culture in SCM, however, NSE was significantly upregulated in all the GBM cell cultures except for A172, in which NSE was downregulated (0.4 ± 0.12, p = 0.002, figure 2A). MAP2 was significantly downregulated in GaMG and LN229 after short-term culture in SCM. After long-term culture it was significantly downregulated in GaMG and pB while upregulated in pA (data not shown). Class III β-tubulin was significantly downregulated in GaMG and U87 while upregulated in LN229 in long-term cultures in SCM (data not shown).

Since NSE was upregulated in SCM, we next investigated NSE expression in short- and long term cultures in serum-starvation medium (figure 2B). NSE was significantly upregulated in all the GBM cell lines for both short-term (fold change in GaMG, 2.3 ± 0.32, p = 0.016; LN229, 1.3 ± 0.01, p < 0.001; U251, 3.2 ± 0.02, p < 0.001; U87, 1.7 ± 0.15, p = 0.009) and long-term culture (fold change in A172, 4.3 ± 1.32, p = 0.046; GaMG, 1.8 ± 0.08, p < 0.001; LN229, 1.9 ± 0.12, p < 0.001; U251, 2.5 ± 0.45, p = 0.014; U87, 1.9 ± 0.32, p = 0.032) except A172 cells cultured for 4 days. Furthermore, NSE expression levels showed only minor differences between short- and long time cultures except for A172, in which NSE was strongly upregulated only in the long-term culture. MAP2 was upregulated in 4 of the 5 GBM cell lines in serum-starvation medium (data not shown).

We also investigated the impact of hypoxia, which has been established as an important factor in promoting tumor aggressiveness and resistance to chemo- and radiotherapy. Thus, we assessed the expression of neuronal markers after 7 days incubation in 0.5% oxygen. NSE was upregulated in all of the 5 GBM cell lines after 7 days in hypoxia, although only LN229 was significant (p = 0.037, figure 2C). All the cells were cultured in serum-containing DMEM medium. MAP2 was downregulated in 4 of the 5 GBM cell lines after 7 days in hypoxia (figure 2C). In contrast to previous studies [18], class III β-tubulin was significantly downregulated in all the cell lines in hypoxia (p < 0.001) except for U87 (figure 2C).

To assess whether the increase of NSE transcripts in gliomas in hypoxia leads to an increase of NSE at the protein level, we analyzed the expression of NSE in GBM cell lines in hypoxia by western blot, normalized by the protein content in normoxia (figure 2D and 2E). The results were in keeping with the findings of real-time qPCR, showing a consistent upregulation of NSE in hypoxia.

### NSE knock-down reduce migration of glioma cells

**Figure 3:**
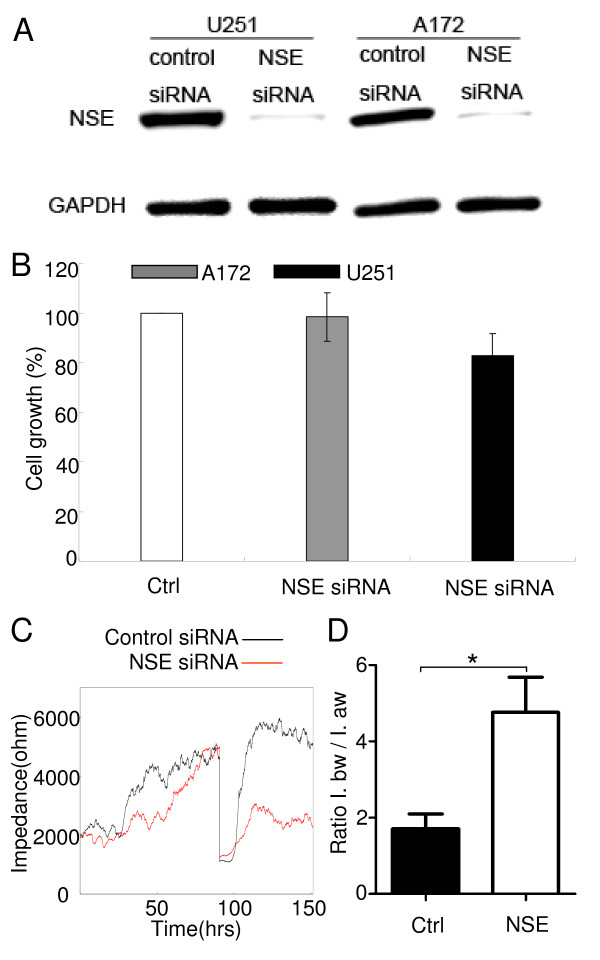
**NSE knockdown reduces the migration of glioma cells**. **(A) **U251 and A172 were transfected with control siRNA or NSE siRNA. NSE protein level was assessed by Western blot. GAPDH was the loading control. **(B) **NSE knockdown in A172 and U251 slightly decreased the proliferation of the cells ( 2-17% ). **(C) **ECIS experiment showed NSE knockdown reduced the migration of A172. **(D) **The ratio of impedance before the wounding and after the wounding until the end of the experiment in both the control and NSE siRNA treatment groups. Error bars represent the SEM. Experiments were performed three times. *P< 0.05. Ctrl, control siRNA.

Since NSE was consistently upregulated in different cellular stress conditions, we hypothesized that it might mediate tumor progression and resistance to various treatment. In order to investigate the role of NSE in glioma cells, we first confirmed knock-down of NSE in glioma cells using synthetic NSE siRNA duplexes (s4685, figure 3A). Knock-down was also observed with another NSE siRNA duplexes (s4684, data not shown). After treatment with NSE siRNA in A172 and U251 for 6 days, approximately 85-95% reduction of the NSE protein was observed on the Western blots.

Compared to control siRNA treatment, NSE siRNA treatment resulted in a slight inhibition of glioma cell growth measured by MTS assay, although this difference was not significant (figure 3B). After treating A172 and U251 with NSE siRNA for 7days, the growth of A172 was inhibited by 2% (p = 0.86), while U251 was inhibited by 17% (p = 0.08). Furthermore, NSE siRNA treatment decreased the migration of A172 cells into areas devoid of cells following wounding experiment with ECIS (figure 3C). Both of the treatment groups had similar proliferation patterns before wounding by electric currents from electrodes underlying the monolayers in the wells. After the wounding however, cells treated with control siRNA migrated faster into the wounded area overlying the electrode compared to cells treated with NSE siRNA, demonstrated by a more rapid increase in impedance with time. There was a significant difference when comparing the ratio of impedance before and after the wounding in the control and knockdown groups (p = 0.038, figure 3D).

### NSE knock-down reduce viability of glioma cells in hypoxia and after irradiation or TMZ-treatment

**Figure 4:**
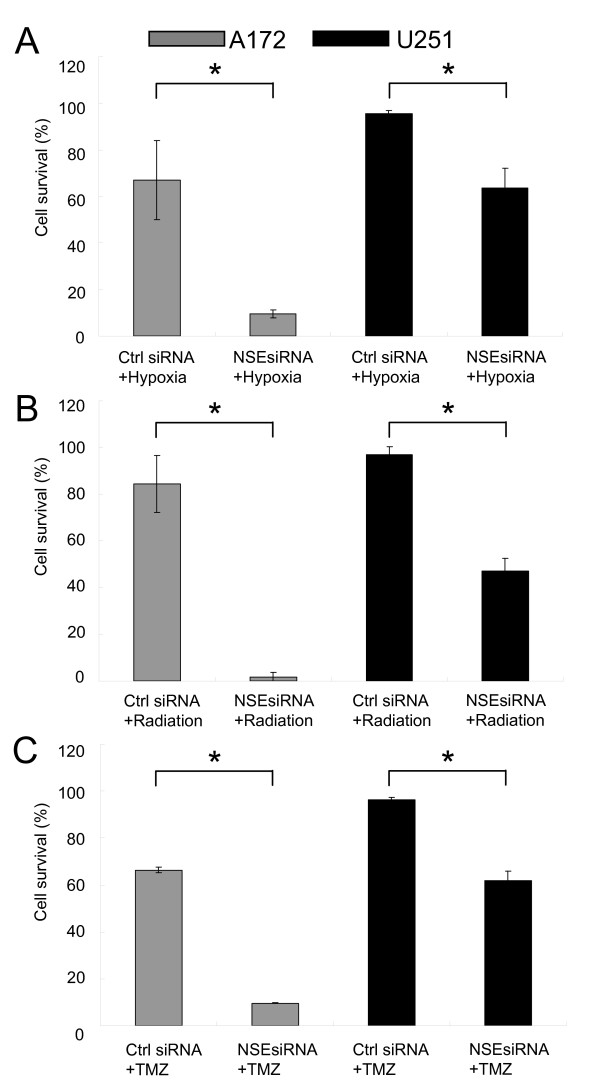
**NSE knockdown sensitizes glioma cells to hypoxia, irradiation and temozolomide**. **(A, B, C) **Glioma cells were plated and transfected by NSE siRNA or control siRNA, then cultured in hypoxia or received 4 Gy irradiation or temozolomide (10μM for A172 & 50μM for U251). Cells transfected by control siRNA without any treatment were used as reference. Error bars represent the SEM. Experiments were performed three times. *P< 0.05. Ctrl, control.

Upon hypoxia treatment, NSE knockdown significantly increased cell death both in the A172 and U251 glioma cell lines as measured by MTS assay (figure 4A). After 6 days incubation in 0.5% oxygen, cell survival dropped to 67.1 ± 17.0% (p = 0.021) for A172 and 95.4 ± 1.7% (p = 0.004) for U251 in the control siRNA treatment group, in the NSE siRNA treatment group cell survival dropped to only 9.5 ± 1.8% (p < 0.001) for A172 and 63.6 ± 8.5% (p < 0.001) for U251. Furthermore, treatment with NSE siRNA also potentiated the effect of 4 Gy irradiation and TMZ treatment on cell death (figure 4B and 4C). After 4 Gy irradiation, cell survival dropped to 84.3 ± 12.2% (p = 0.090, A172) and 97.0 ± 3.4% (p = 0.220, U251) in the control siRNA treatment group while in the NSE siRNA treatment group cell survival dropped to only 1.6 ± 2.1% (p < 0.001, A172) and 47.2 ± 5.4% (p < 0.001, U251). After TMZ treatment for 5 days, cell survival dropped to 66.4 ± 1.1% (p = 0.011, A172) and 96.3 ± 1.0% (p < 0.001, U251) in the control siRNA treatment group, when pretreated with NSE siRNA, cell survival dropped to only 9.6 ± 0.2% (p < 0.001, A172) and 61.8 ± 4.0% (p < 0.001, U251).

### NSE is expressed in a panel of human glioma biopsies

**Figure 5:**
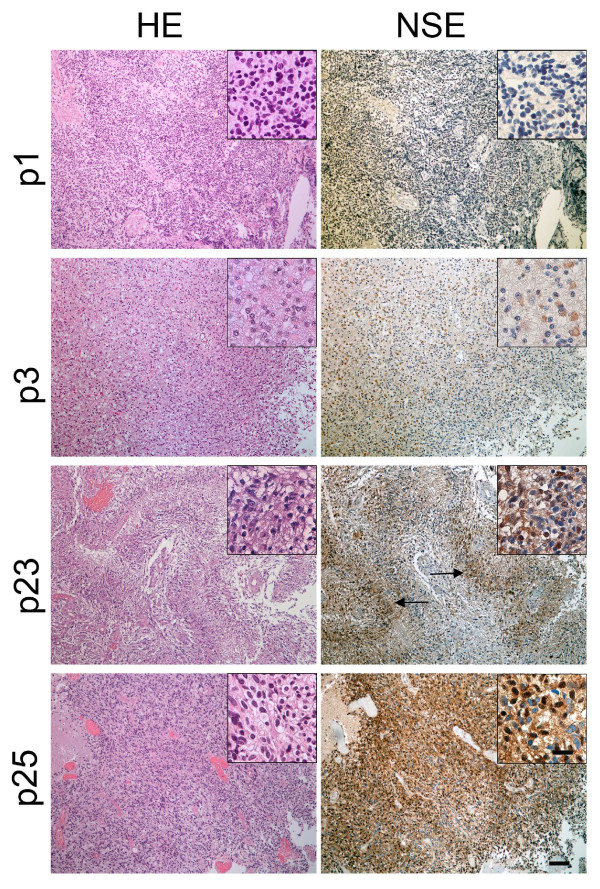
**NSE expression in human glioma specimens**. HE staining and ICH staining for NSE on sections from patient specimens. p1 and p3 belonged to the NSE low expression group while p23 and p25 belonged to the NSE high expression group. p3 was oligoastrocytoma (grade II), p1, p23 and p25 were GBMs. NSE staining existed both in the nucleus and cytoplasma. Arrows represent pseudopalisading cells surrounding areas of necrosis. Scale bars in the low magnification image (10×) = 100 μm, in the high magnification image (40×) = 25 μm.

In order to assess the clinical relevance of our findings, we performed IHC on a panel of 28 consecutively harvested biopsies from a cohort of glioma patients operated at our hospital. Gliomas of all grades stained positive for NSE, although the staining intensity and pattern varied markedly among patients. The mean area fraction of immunopositivity was 5.7% for all gliomas, varying between 0.3% and 30.7%. All tumors exhibited both nuclear and cytoplasmic stainings (figure 5). NSE staining was detected both in the vascular and necrotic areas, however, increased cytoplasmic and nuclear stainings were observed in pseudopalisading cells surrounding areas of necrosis (figure 5, p23).

### High NSE expression is associated with shorter survival in GBM patients

**Figure 6:**
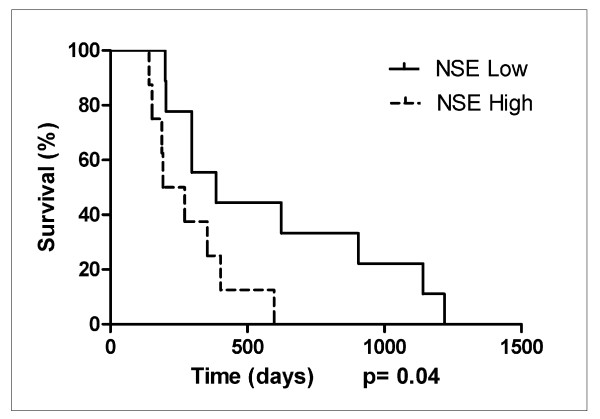
**High NSE expression is associated with shorter survival in GBM patients**. Kaplan-Meier survival analysis of GBM patients grouped by NSE expression. Among GBM patients receiving standard radiotherapy treatment (n = 17) there was a significant difference in survival (P = 0.04) between patients with low vs. high NSE expression.

We obtained clinical data for the patients from whom the biopsies were obtained, and found that median survival was 297 days for the GBM patients and 370 days for the whole group of patients with both low and high grade gliomas, with 4 patients being alive at the end of the study. Next, we wanted to determine whether NSE expression impacted on the survival outcome in the patients diagnosed with GBM. Patients were ranked by the area fraction staining positive for NSE in the biopsy tumor sections and grouped accordingly, into two equally sized groups of 10 patients with lower and 11 patients with higher NSE expression. Kaplan-Meier analysis demonstrated that GBM patients in the group with high NSE expression lived significantly shorter (192 vs. 341 days, p = 0.04). Moreover, when excluding the 4 patients who did not receive the standard treatment of 54 Gy or more, the difference remained significant between the 9 patients in the low expression group and the 8 patients in the high expression group. Patients with low and high NSE expression had a median survival of 385 and 232 days, respectively (p = 0.04, figure 6). To rule out that this was caused by our specific cut-off, we regrouped the patients so that the low expression group contained 8 patients and the high expression group contained 9 patients. However, we still found a significantly longer survival for patients with low vs high NSE expression (505 vs. 200 days, p = 0.01).

## Discussion

Enolase is one of the glycolytic enzymes, and exists as a dimer of two subunits. There are three kinds of subunits, α-, β- and γ- subunits. NSE is a γγ- isozyme, also called ENO2, and was reportedly only detected in neurons and neuroendocrine cells under physiological conditions [19,20]. Glycolysis is considered the main source of energy for cancer cells [21]. Interestingly, astrocytic tumor cells also stain positively for NSE [22], suggesting that NSE-negative cells can acquire the ability to produce NSE. This may reflect a form of adaptation to the increased metabolic demands associated with a neoplastic state [23]. Furthermore, others have reported that NSE has a neurotrophic and neuroprotective effect on neurons in the CNS mediated by specific binding to the neuronal surface [24]. Thus, NSE may also display non-glycolytic functions, possibly acting as a survival factor. Concordant with previously reported studies [22,25], we found that NSE was aberrantly expressed in all the five GBM cell lines and two patient biopsies, and was upregulated both in serum-starvation medium and under hypoxic conditions. Thus, NSE may play a role when glioma cells adjust to a niche dominated by hypoxia and lack of nutrients as tumors outgrow their blood supply. Interestingly, NSE was also upregulated in long-term cultures in SCM.

Moreover, the involvement of NSE in glycolysis and its role as a possible survival factor may also imply that it represent a therapeutic target [26]. Indeed, knockdown of NSE inhibited the proliferation by 17% in U251, although not significantly (p = 0.08), NSE knockdown also reduced the migration of A172 in the wounding experiments. Furthermore, NSE knockdown significantly reduced the viability of GBM cell lines in hypoxia. Since hypoxia, has been linked to chemo- and radioresistance [27], overall tumor aggressiveness, as well as upregulation of NSE in our experiments, it also suggests that expression levels of NSE may correlate with prognosis in glioma patients. Notably, overexpression of ENO1, another enolase isozyme, in hepatocellular carcinoma [28] and head and neck cancer [29] has been associated with poor clinical outcomes. In our stuy, glioma patients displaying higher NSE expression levels had a significantly shorter survival. Our findings are in accordance with a previous study [5]. This may result from the effect of NSE upon the response to radiotherapy and chemotherapy, as we found that knockdown of NSE sensitized these GBM cell lines to irradiation and TMZ. It should be emphasized however, that the mean age in the NSE low expression group was lower than in the NSE high expression group (52.5 vs 64.5). Moreover, 4 patients in the NSE high expression group did not receive chemotherapy. As such, our study suffers from the same limitations as other retrospective studies, with a biased selection of patients which influences the results. Therefore, controlled studies will be needed to validate the clinical significance of NSE in a greater number of glioma patients. In fact, the expression and prognostic value of NSE has been investigated in several other tumors, including non small cell lung cancer [30], breast cancer [31] and prostate cancer [32]; however, the expression of NSE in lung cancer and breast cancer is associated with better survival, while NSE staining was associated with shorter survival [32] or was of no practical value as an independent prognostic indicator in patients with prostate cancer [33]. Vos et al [34] also reported there was no prognostic value of serum NSE levels in brain tumor patients.

Due to the cytoplasmic staining of NSE, it is difficult to identify single positive cells in the field. For this reason, we applied the area fraction of immunopositivity method as previously described [16,35]. Previously, we have used this method to obtain accurate estimates of immunopositivity with cytoplasmic stainings [16].

In our study, both the hmw-MAP2 and lmw-MAP2 could be detected in GBM cells. However, different quantities of the isoforms were expressed in different cell lines. Interestingly, different expression levels were noted under different culture conditions. Class III β-tubulin was significantly downregulated in four GBM cell lines in hypoxia, which contrasts previous studies. Previously, increased expression of class III β-tubulin has been observed in GBMs bordering geographic areas of ischemic necrosis [8,36], the discrepancy between in vivo and in vitro observations warrants further investigation.

## Conclusions

This study shows that neuronal markers are aberrantly expressed both in GBM cell lines and patient biopsies. Furthermore, their expressions are altered by cellular stress. NSE is consistently upregulated in different cellular stress conditions. NSE knockdown potentiates the effect of chemo- and radiotherapy and expression levels are inversely associated with survival. Further studies are needed to investigate whether this can be explored therapeutically.

## Competing interests

The authors declare that they have no competing interests.

## Authors' contributions

TY carried out all of the experiments except for ECIS and wrote this manuscript. KOS helped qPCR and contributed to data analysis. LL performed cell culture, including hypoxia experiments, immunocytochemistry, RNA isolation and Western blot. LS carried out ECIS experiment. JW, XL and PØE participated in study design and manuscript preparation. All authors read and approved the final manuscript.

## Pre-publication history

The pre-publication history for this paper can be accessed here:

http://www.biomedcentral.com/1471-2407/11/524/prepub
